# Research on Configuration Design Optimization and Trajectory Planning of Manipulators for Precision Machining and Inspection of Large-Curvature and Large-Area Curved Surfaces

**DOI:** 10.3390/mi14040886

**Published:** 2023-04-20

**Authors:** Xiangyang Sun, Shuai He, Zhenbang Xu, Enyang Zhang, Yanhui Li

**Affiliations:** 1 Changchun Institute of Optics, Fine Mechanics and Physics, Chinese Academy of Sciences, Changchun 130033, China; sunxiangyang19@mails.ucas.ac.cn (X.S.); zhangenyang@ciomp.ac.cn (E.Z.); liyanhui@ciomp.ac.cn (Y.L.); 2 University of Chinese Academy of Sciences, Beijing 100049, China; 3 CAS Key Laboratory of On-Orbit Manufacturing and Integration for Space Optics System, Chinese Academy of Sciences, Changchun 130033, China; 4 Center of Materials Science and Optoelectronics Engineering, University of Chinese Academy of Sciences, Beijing 100049, China

**Keywords:** redundant manipulator, large curvature, large area surface, configuration optimization, trajectory planning

## Abstract

In recent years, high-quality surfaces with large areas and curvatures have been increasingly used in engineering, but the precision machining and inspection of such surfaces is a particular challenge. Surface machining equipment needs to have a large working space, high flexibility, and motion accuracy to meet the demands of micron-scale precision machining. However, meeting these requirements may result in extremely large equipment sizes. To solve this problem, an eight-degree-of-freedom redundant manipulator with one linear and seven rotational joints is designed to assist in the machining described in this paper. The configuration parameters of the manipulator are optimized by an improved multi-objective particle swarm optimization algorithm to ensure that the working space of the manipulator completely covers the working surface and that the size of the manipulator is small. In order to improve the smoothness and accuracy of manipulator motion on large surface areas, an improved trajectory planning strategy for a redundant manipulator is proposed. The idea of the improved strategy is to pre-process the motion path first and then use a combination of the clamping weighted least-norm method and the gradient projection method to plan the trajectory, while adding a reverse planning step to solve the singularity problem. The resulting trajectories are smoother than those planned by the general method. The feasibility and practicality of the trajectory planning strategy are verified through simulation.

## 1. Introduction

With the rapid development of science and technology, many mechanical products are being updated, and the requirements for large surfaces with large curvature in mechanical products are becoming more and more demanding, such as in the head shells of aircraft, rockets, and moving vehicles. In order to improve the quality and production efficiency of curved surfaces, robots have been widely used in processes such as grinding, polishing, painting, and inspection of high-quality curved surfaces [1,2,3,4,5,6,7].

There are two prerequisites for robots to be able to perform the task of assisting in the micron-scale precision machining of surfaces. One is that the working space of the robot covers the working surface, and the other is a suitable surface movement trajectory. The working space of the robot has a great relationship with the structure of the robot. Nowadays, robots commonly used for surface machining include SCARA robots and five- and six-axis industrial robots, which are mainly used for flat machining and small-curvature surface machining [8,9,10]. Some researchers have investigated parallel and hybrid robots, which have the advantage of a large working space and good stiffness. However, their disadvantage is that the robots are large and costly and need to work in a specific workplace [11,12,13,14,15,16]. So, it is necessary to design a manipulator for surface machining with a large working space and a relatively small size.

To ensure the quality of the surface, the trajectory of the manipulator needs to be smooth when moving on the surface, and the common method for manipulator trajectory planning is to interpolate the trajectory points using straight lines, circular arcs, polynomial curves, B-spline curves, S-curves, and so on [17,18,19]. Zhang Peng et al. studied a large curvature surface spraying robot and its spraying path planning, combined with the idea of a particle swarm optimization algorithm, and wrote an algorithm suitable for the study of large curvature surface spraying trajectory sequencing and combination problems [20,21], but the spraying area of the robot they studied was less than 1 m^2^. Shao Junyi et al. studied a redundant robot for pipe inner wall spraying. A redundant robot trajectory planning method was established for general spatial curved pipe-type parts inner surface spraying operations [22]. These surface trajectory planning methods are mainly applicable to small surfaces; however, the complexity and length of the trajectory increase significantly when machining large-curvature surfaces with large areas. This requires a trajectory planning method that can quickly plan a long, continuous, and smooth trajectory, but the results obtained by general trajectory planning methods are often unsatisfactory.

This paper designs an eight-degree-of-freedom redundant surface scanning manipulator. A multi-objective particle swarm optimization algorithm is used to optimize its configuration, resulting in a large available working space and a relatively small size. A surface trajectory planning algorithm combining the pincer-weighted minimum parametric method and the projected gradient method is proposed to improve the continuity and smoothness of the manipulator trajectory, and the algorithm is verified by simulation and experiment.

## 2. Configuration Design and Optimization

### 2.1. Configuration Design

**Figure 1:**
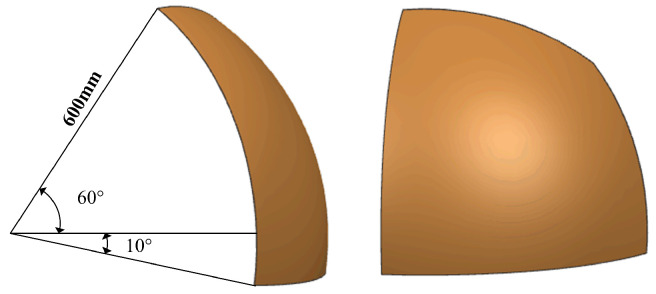
Working surface.

**Figure 2:**
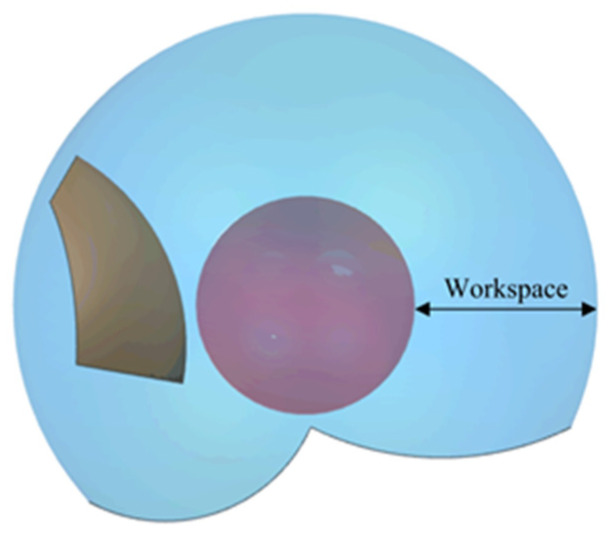
Workspace of industry robot.

When working on large surfaces with large curvature, the working space of the manipulator must be able to cover the surface completely, and the manipulator must have as few singularities as possible when moving on the surface. It is also important to consider the utilization of the working space. In general, the working space of a robot is similar to a hollow sphere, so if a large convex surface is to be covered completely, a large part of the working space will be left unused. As an example, take a shaped spherical surface of large curvature with a radius of 600 mm and an angle of −10° to 60°. The shape of the surface is shown in Figure 1. A typical six-degree-of-freedom industrial robot has a workspace similar to a notched hollow sphere, as shown in Figure 2, and a large part of its workspace will be unused when it completely covers the working surface.

**Figure 3:**
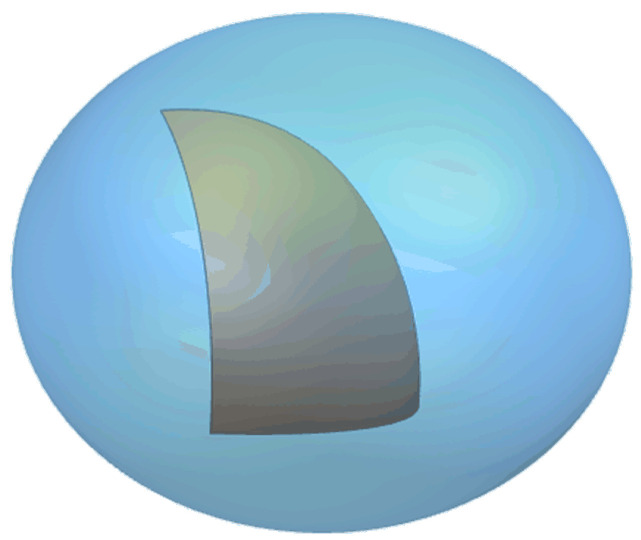
Workspace of eight-degree-of-freedom redundant manipulator.

In this paper, an eight-degree-of-freedom redundant manipulator is designed to meet the working requirements. The structure of the manipulator is designed with one horizontal moving joint and seven rotating joints. The seven rotating joints increase the flexibility of the manipulator, allowing it to reach all positions on the machined surface. The horizontal moving joint extends the working space of the manipulator into an ellipsoidal shape, increasing the utilization of the working space. The working space of the manipulator designed in this paper is shown in Figure 3.

**Figure 4:**
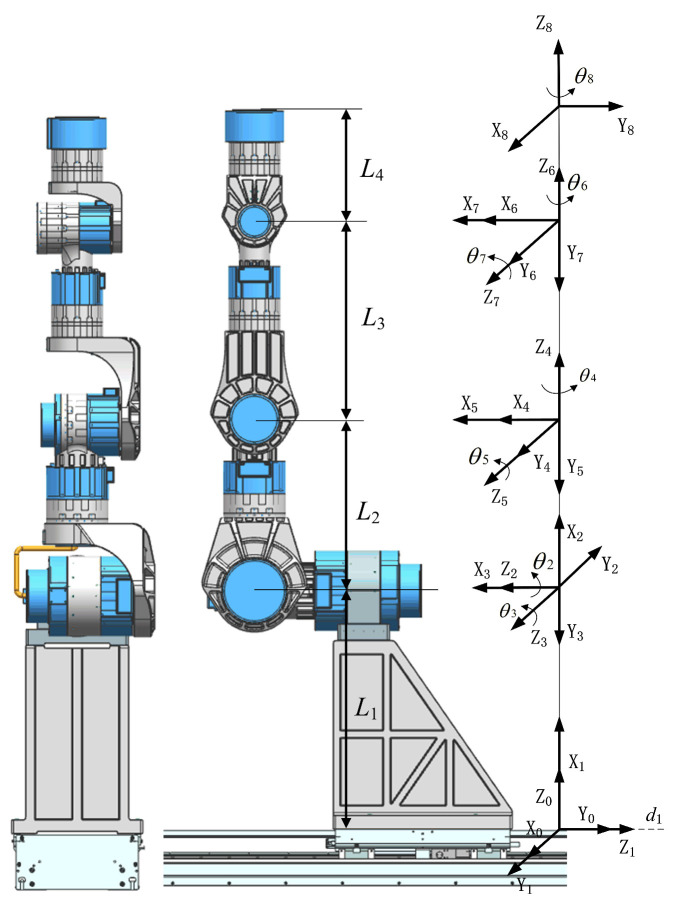
Configuration of the manipulator.

The configuration of the manipulator and the linkage coordinate system are shown in Figure 4. The D-H parameters of the manipulator are shown in Table 1. DH parameters are the most common and concise way to determine the manipulator configuration, and the accuracy of DH parameters has a great impact on the motion planning of the manipulator. Structural optimization of the manipulator means optimization of DH parameters. Moreover, the DH parameters can be set and calibrated to reduce the impact of assembly errors on the accuracy of the manipulator [23]. In Table 1, *L*_1_ is the distance from the rotating joint to the moving joint, and *L*_2_, *L*_3_, and *L*_4_ are the linkage lengths of the manipulator. The lengths of *L*_2_ and *L*_4_ are determined according to the requirements of stiffness, joint structure, and load-bearing capacity, while the lengths of *L*_1_ and *L*_3_ have to be optimized to obtain suitable values according to the working requirements.

### 2.2. Configuration Optimization

The working space of the manipulator is directly related to the length of the linkage. Meanwhile, the structural stiffness of the manipulator is negatively related to the length of the linkage; the longer the linkage, the less stiff the manipulator. Therefore, there are two objectives for optimizing the manipulator configuration. One is to satisfy the requirement that the working space completely covers the working surface and that the manipulator moves on the surface with fewer singularities. The other is to keep the overall length of the manipulator as short as possible to reduce its weight and increase its stiffness. To find out the optimum configuration that meets the requirements, the manipulator configuration parameters *L*_1_ and *L*_3_ need to be optimized according to the working surface.

The most common optimization algorithms are genetic algorithms, ant colony algorithms, neural network algorithms, and particle swarm algorithms [24,25,26,27]. The particle swarm optimization algorithm (PSO) is a heuristic algorithm inspired by bird flock choreography. The basic principle of particle swarm optimization algorithms is to treat the solution to an optimization problem as a particle in the search space, with each particle having a fitness value determined by the objective function. The size of the fitness value determines whether the particle is superior or inferior. Each particle also has a search speed that determines the direction and distance of the search, which is related to the historical optimal value of the particle itself and the overall optimal value of the particle population. The optimal solution to the optimization problem can be obtained after a certain number of iterations.

Particle swarm algorithms have been found to be successful in a variety of optimization problems. The particle swarm algorithm performs well in single-objective optimization problems and converges very quickly [28,29,30]. Coello et al. proposed a multi-objective particle swarm optimization algorithm, confirming the feasibility and superiority of particle swarm algorithms for multi-objective optimization problems [31]. The multi-objective swarm optimization algorithm solves an optimization problem with k objective functions, which means that the fitness value of each particle is a k-dimensional vector, and it is not possible to simply compare and determine whether the particles are superior or inferior. Therefore, the multi-objective swarm optimization algorithm uses Pareto Dominance, which is defined as follows: A vector $\vec{u}=(u_{1},u_{2},\ldots u_{k})$ is said to dominate $\vec{v}=(v_{1},v_{2},\ldots v_{k})$ (denoted by $\vec{u}\underline{≺}\vec{v}$) if and only if u is partially less than v, i.e.,

(1) $$\forall i\in \left\{1,\ldots k\right\}:u_{i}\leq v_{i}\wedge \exists i\in \left\{1,\ldots k\right\}:u_{i}<v_{i}$$

The global optimal solution of a multi-objective particle swarm optimization algorithm is a set, and the solutions in the solution set are all non-dominated optimal solutions in the sense that none of them will be dominated by the other solutions. The global optimal solution is selected by roulette from the set of global optimal solutions when updating the particle velocity.

In this paper, the multi-objective particle swarm optimization algorithm is used to optimize the configuration of the manipulator. The elements in the particles are the lengths of the linkages *L*_1_ and *L*_3_, and the range of values of *L*_1_ and *L*_3_ is the search space of the optimization problem. The total length of the manipulator is used as the first objective function. The number of singularities encountered during the trajectory planning process is used as the second objective function. Each particle has fitness values *F_1_* and *F_2_* determined by the two objective functions. The range of values for *L*_1_ and *L*_3_ in the multi-objective swarm optimization algorithm is a large range estimated according to the design criteria, so some particles may encounter no solutions for the trajectory points during the optimization process, i.e., invalid solutions. If these invalid particles are removed without processing, other particles may still become invalid during the search, which will greatly reduce the convergence speed. In order to solve the problem of slow convergence due to the wide range of particles, this paper proposes an improved optimization algorithm combining the particle swarm algorithm and the artificial potential field method. The improved algorithm is divided into two steps. The first step is to carry out a smaller number of iterations of optimization. In the optimization process, when the invalid particles are encountered, they will be considered obstacles. The exclusion potential field will be set at the obstacles, giving the particles in the vicinity of the invalid solution a speed away from the obstacles. The search speed of the particles is increased, and the entire search space is searched quickly. At the same time, all the invalid solutions are recorded to form the invalid solution set. After the optimization is complete, a more accurate range of particle values can be derived from the distribution of particles in the invalid solution set. Then, in the second optimization step, the range of particle values is changed to the range obtained in the first step so that the probability of encountering invalid solutions is greatly reduced. The invalid solution set is also substituted as an obstacle in the second step of the algorithm so that the particles can keep away from the invalid solution area, thus speeding up the convergence.

**Figure 5:**
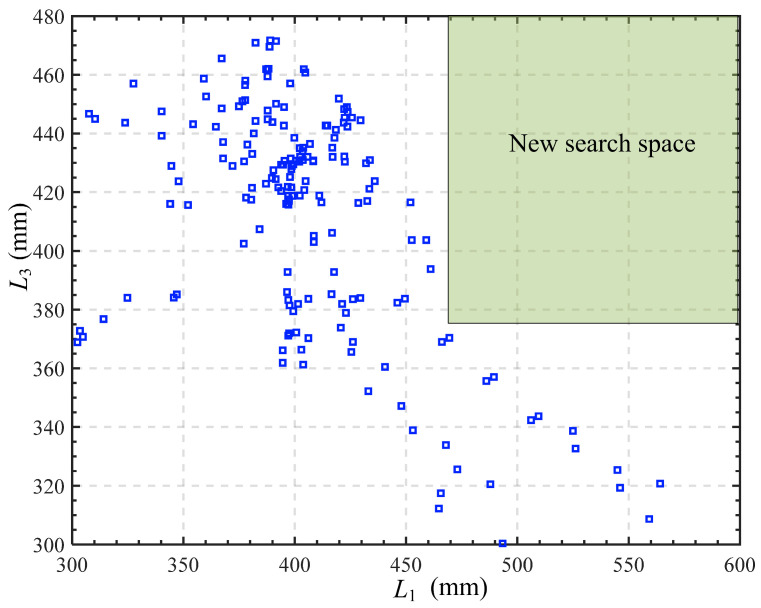
The result of the first step of optimization.

**Figure 6:**
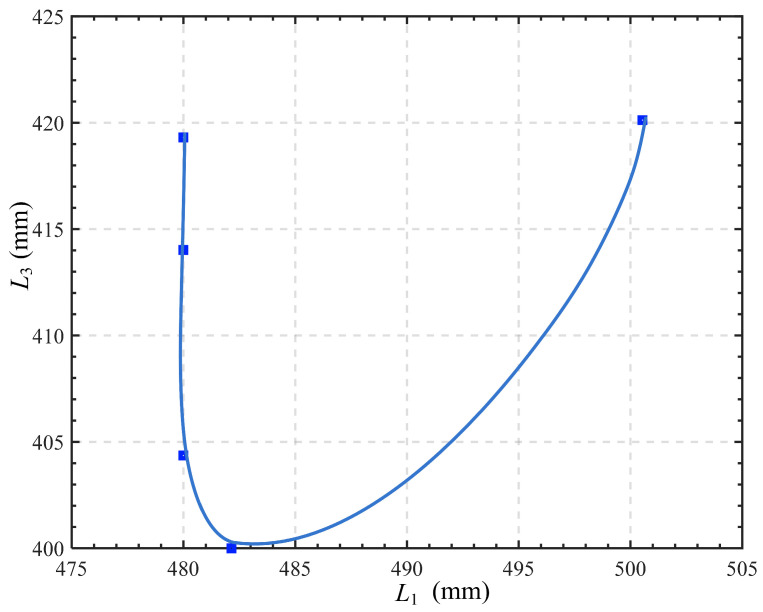
The result of the second step of optimization.

The improved multi-objective particle swarm optimization algorithm proposed in this paper is used to optimize the lengths of the linkages *L*_1_ and *L*_3_. The initial range of values of *L*_1_ and *L*_3_ is given as $L_{1}\in (300,600)$, $L_{3}\in (300,500)$. The set of invalid solutions is obtained by the first step of the improved algorithm, as shown in Figure 5, and according to the region where the invalid solutions are located, the new range of values for *L*_1_ and *L*_3_ is determined as $L_{1}\in (480,600)$, $L_{3}\in (380,480)$. The invalid solution is then substituted as an obstacle in the second optimization step, and the result obtained from the optimization is shown in Figure 6. Finally, the values of *L*_1_ and *L*_3_ are determined to be $L_{1}=480$ and $L_{3}=418$.

## 3. Trajectory Planning

### 3.1. Trajectory Planning Method 

Trajectory planning is the calculation of the velocity and acceleration of the manipulator based on the motion path, so that the motion of the manipulator is smoother and the drastic changes in velocity and acceleration are reduced [8,9,10]. The trajectory planning of the manipulator, whether in joint space or in Cartesian space, is to first calculate the joint angle corresponding to the pose at the path point in the path, and then interpolate the joint angle to obtain the joint angular velocity and angular acceleration.

The manipulator designed in this paper is a redundant manipulator, and one end pose of the redundant manipulator corresponds to an infinite number of inverse solutions. It means that it is difficult to derive an analytical inverse solution, so the numerical solution method is usually used. The numerical solution method is to use the pseudo-reverse of the Jacobian matrix to find the inverse solution [11]. At the same time, the range of joint angles should be taken into account. The most common numerical methods for solving inverse kinematics considering angle constraints are the gradient projection method and the weighted least-norm method [32,33]. The gradient projection method exploits the existence of a null-space for a singular Jacobi matrix to achieve joint angle restriction by minimizing the penalty function along the gradient direction in the Jacobi null-space of the master task. Null-space means that any angular velocity vector in this space multiplied by the Jacobi matrix results in a zero vector. In other words, the end pose of the manipulator does not change when the joints of the manipulator move according to the angular velocity vector in null-space. The gradient projection method is defined as

(2) $$\dot{\theta }=J^{+}\dot{x}+(I_{n}-J^{+}J)H$$

where $\dot{x}$ is the end velocity of the manipulator as a 6-dimensional vector, $\dot{\theta }$ is the angular velocity of each joint as an *n*-dimensional vector, *n* represents the number of degrees of freedom of the manipulator, J represents the Jacobi matrix, and $J^{+}$ represents the pseudo-reverse of the Jacobi matrix. $I_{n}$ is a unit matrix of order *n*, $I_{n}-J^{+}J$ is the null-space projection operator of the Jacobian matrix, $H\in R^{n}$ is an arbitrary *n*-dimensional vector, and $(I_{n}-J^{+}J)H$ can be used to perform sub-tasks such as joint limiting.

The weighted least-norm method limits the joint angles by adding weight factors to the joint velocities. As the joints approach their limits, the corresponding joints are set with smaller weight factors, effectively suppressing the operating velocities of the corresponding joints. The weighted least-norm method is effective in reducing invalid self-motion and is therefore more efficient than the gradient projection method. The weighted minimum parametric method is defined as

(3) $$\dot{\theta }=W^{-1}J^{T}(JW^{-1}J^{T}+\lambda^{2}I_{m}{)}^{-1}\dot{x}$$

where W denotes the weight matrix and the weight matrix is an *n*-order diagonal array, each diagonal element is the weight of each joint angle, and $\lambda^{2}$ is the damping factor to help deal with the singularity of the manipulator [33].

Once the inverse solution for all path points is obtained, the angles of the joints can be interpolated according to the movement time of the manipulator, typically using straight lines, circular arcs, polynomial curves, B-spline curves, S-curves, etc. After interpolation, the velocity and acceleration of the manipulator can be calculated by differentiation. The result of the trajectory planning is highly dependent on the kinematic inverse solution; if there are more drastic changes in the inverse solution, it will lead to drastic changes in the velocity and acceleration of the manipulator, making the trajectory of the manipulator discontinuous.

### 3.2. An Improved Trajectory Planning Strategy

The manipulator studied in this paper is used on a large curvature and a large convex surface, which has a very long moving trajectory during operation. In the trajectory planning process, there is a high probability of encountering singularities that lead to drastic changes in joint angles. Therefore, this paper proposes an improved trajectory planning strategy by segmenting the motion path and setting up self-motion areas at the edges of the path. The inverse solution of the path points is then found based on a combination of the clamping weighted least-norm method and the projected gradient method, and the singular points are moved to the self-motion area by reverse planning when the singular points are difficult to solve by the projected gradient method. By increasing the motion time in the self-motion area, the joint angle changes are not as drastic, and the motion along the working path is guaranteed to be smooth and continuous.

#### 3.2.1. Pre-Processing Motion Path

**Figure 7:**
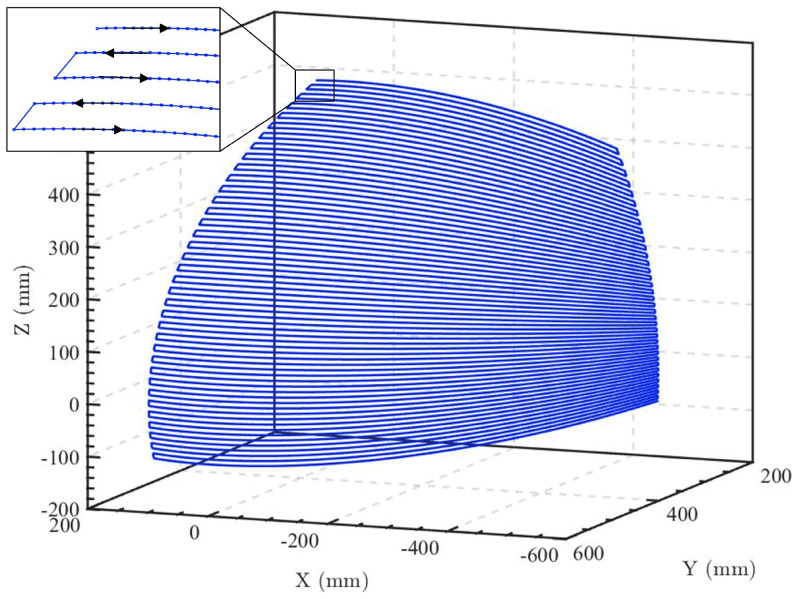
Motion path.

**Figure 8:**
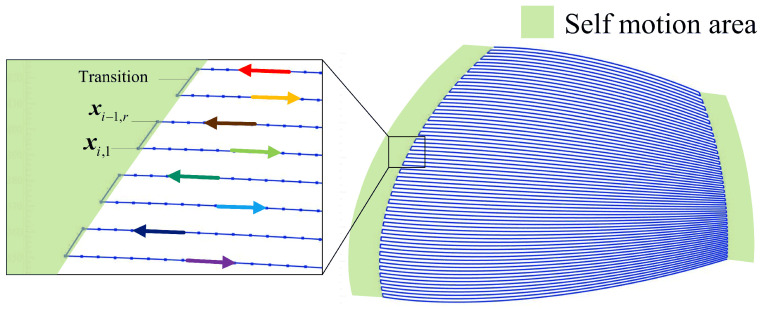
Processed motion path.

Using the local spherical surface described in Section 2 as an example, the path planning method used to machine this surface is to discretize the surface into *P* path points and then connect all the path points in series with an s-shaped reciprocal curve. The paths are shown in Figure 7. The improved trajectory planning strategy proposed in this paper requires pre-processing of the paths. The first step is to segment the path into *r* rows, with *c* path points in each row. Next, the path points on each segmented path are given a two-dimensional coordinate shaped as $x_{i,j}$, where *i* represents the *i*-th segment and *j* represents the *j*-th path point of that segment, and the next point at the end of each segmented path is the starting point of the next segmented path. Finally, the transition between segments and the region at the edge of the path is set as the self-motion area. The pre-processed motion path is shown in Figure 8.

#### 3.2.2. Trajectory Planning Strategy

This paper uses a combination of the clamping weighted least-norm method and the projected gradient method to find the inverse of the path point. The clamping weighted least-norm method is based on the weighted least-norm method with the addition of a clamp term $\left(I_{n}-\tilde{W})\phi (\theta \right)$, which has the advantage of helping the joint to move away from the joint limit as soon as possible when the joint approaches the angle limit [34]. The clamping weighted least-norm method is defined as

(4) $$\dot{\theta }=-(I_{n}-\tilde{W})\phi (\theta )+\tilde{W}J^{T}(J\tilde{W}J^{T}+\lambda^{2}I_{m}{)}^{-1}((x_{i+1}-x_{i})+J(I_{n}-\tilde{W})\phi (\theta ))$$

where $\tilde{W}$ represents the weighting and ϕ(θ) represents the clamp task.

The specific steps of trajectory planning are as follows: (1) Solve the inverse of the poses of all path points in order according to the determined path coordinates, and use the inverse solution $\theta_{i,j}$ of the poses of path point $x_{i,j}$ as the iterative initial value when solving for the inverse solution $\theta_{i,j+1}$ of the poses of path point $x_{i,j+1}$. The iterative solution is carried out by the clamping weighted least-norm method, and the calculation process is as follows:

(5) $$\left\{\begin{array}{c} x_{e}=\mathrm{forward}\_\mathrm{kinematics}(\theta_{i,j})\\ \begin{array}{cc} \dot{\theta }= & -(I_{n}-\tilde{W})\phi (\theta_{i})+\tilde{W}J^{T}(J\tilde{W}J^{T}+\\ & \lambda^{2}I_{m}{)}^{-1}((x_{i,j+1}-x_{e})+J(I_{n}-\tilde{W})\phi (\theta_{i,j})) \end{array}\\ \theta_{i,j+1}=\theta_{i,j}+\dot{\theta }d_{t}\\ x_{e}=\mathrm{forward}\_\mathrm{kinematics}(\theta_{i,j+1})\\ error=x_{i,j+1}-x_{e}\\ \theta_{i,j}=\theta_{i,j+1} \end{array}\right.$$

where *d_t_* represents the step length and error represents the error between the current pose and the desired pose. When it is less than the given accuracy requirement, the inverse solution $\theta_{i,j+1}$ of the pose of the path point $x_{i,j+1}$ is obtained.

(2) Determine whether the inverse solution $\theta_{i,j+1}$ of path point $x_{i,j+1}$ is singularity (based on the distance between the path points and the maximum angular velocity of the manipulator joint, the maximum value of the difference between the inverse solutions of the two adjacent points is greater than 50° as the basis for determining whether it is singularity). If it is singularity, the manipulator is first made to self-motion using the projected gradient method, so that the inverse solution $\theta_{i,j+1}$ of path point $x_{i,j+1}$ changes without changing the end pose, reducing the difference between it and the inverse solution $\theta_{i,j}$ of path point $x_{i,j}$, and eliminating as much as possible the drastic change in joint angle. If the difference between the inverse solutions is less than 50° after the self-motion, proceed to step 4, otherwise, proceed to step 3.

(3) Randomly solve for path point $x_{i,j+1}$ to obtain fifty $\theta_{i,j+1}$ as initial values and carry out reverse planning. Reverse planning is to follow steps 1 and 2 to find the inverse solution of the path points starting at $x_{i,j+1}$ and ending at $x_{i,1}$. When the inverse solution from $x_{i,j+1}$ to $x_{i,1}$ is completed for an initial value of $\theta_{i,j+1}$, reverse planning is stopped, the inverse solution from $\theta_{i,j+1}$ to $\theta_{i,1}$ is replaced with the new one, and trajectory planning continues from $x_{i,j+1}$. In this way, the singularity is transferred to $x_{i-1,r}$. Although the difference between $\theta_{i,1}$ and $\theta_{i-1,r}$ becomes larger, the large incremental change between $\theta_{i,1}$ and $\theta_{i-1,r}$ can be accomplished by self-motion in the self-motion area. In this way, the singularities are resolved outside the working surface, and the smooth trajectory of the manipulator on the working surface can be guaranteed. If all the initial values are unsuccessful in reverse planning, then $x_{i,j+1}$ is marked as a singularity, and a large incremental change between $\theta_{i,j}$ and $\theta_{i,j+1}$ can then be achieved by sacrificing some accuracy in the motion. The method is to choose $\theta_{i,j+1}$, which has the smallest difference with $\theta_{i,j}$ in the process of self-motion, as the inverse solution of $x_{i,j+1}$. Firstly, the manipulator is moved away from the working surface by the horizontal moving joint, then the remaining seven rotation joints are moved from $\theta_{i,j}$ to $\theta_{i,j+1}$, and finally the horizontal moving joint is moved to $\theta_{i,j+1}$. This will make the end of the manipulator out of alignment with the working surface in the process of moving from $x_{i,j}$ to $x_{i,j+1}$, but it ensures that the trajectory of the manipulator passes through all the path points accurately.

(4) Determine whether path point $x_{i,j+1}$ is the last path point; if it is, then proceed to step 5; otherwise, determine whether *j* + 1 is equal to *c*; if it is, then make *i* = *i* + 1 and proceed to step 1, if not, then make *j* = *j* + 1 and proceed to step 1.

(5) Interpolate the resulting joint angle in the joint space with a quintic polynomial to obtain the joint angular velocity and angular acceleration of the manipulator, and the trajectory planning is completed.

**Figure 9:**
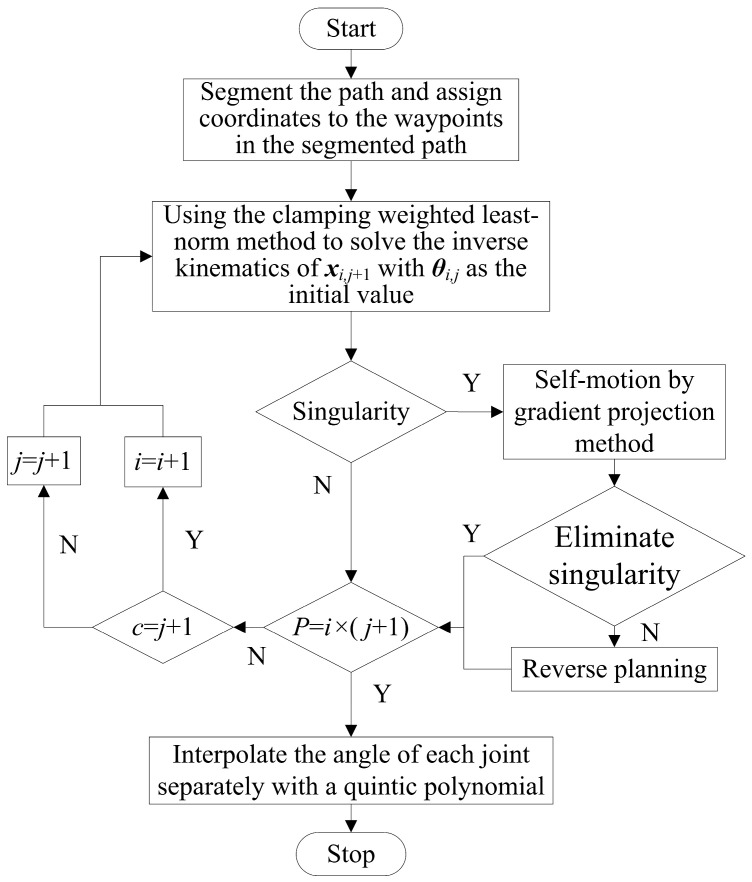
Trajectory planning strategy flowchart.

The overall flow of the trajectory planning strategy is shown in Figure 9.

## 4. Simulation and Experimentation

**Figure 10:**
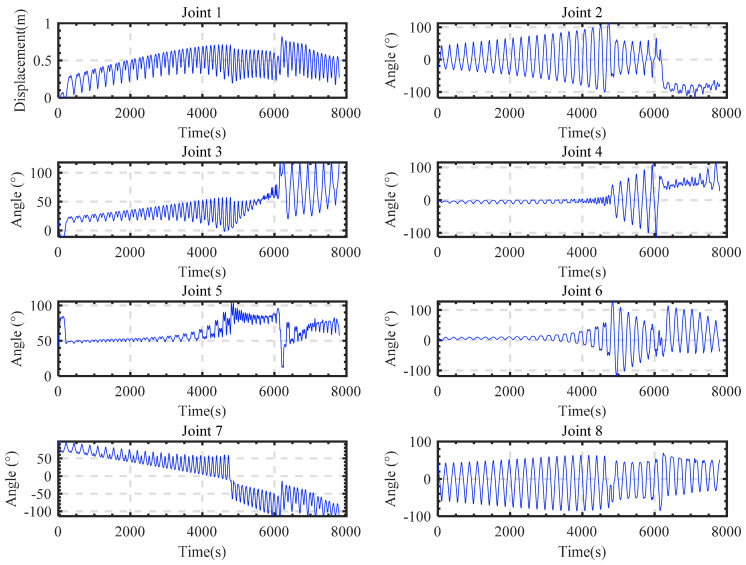
The angle of each joint of the trajectory planned by the improved trajectory strategy.

**Figure 11:**
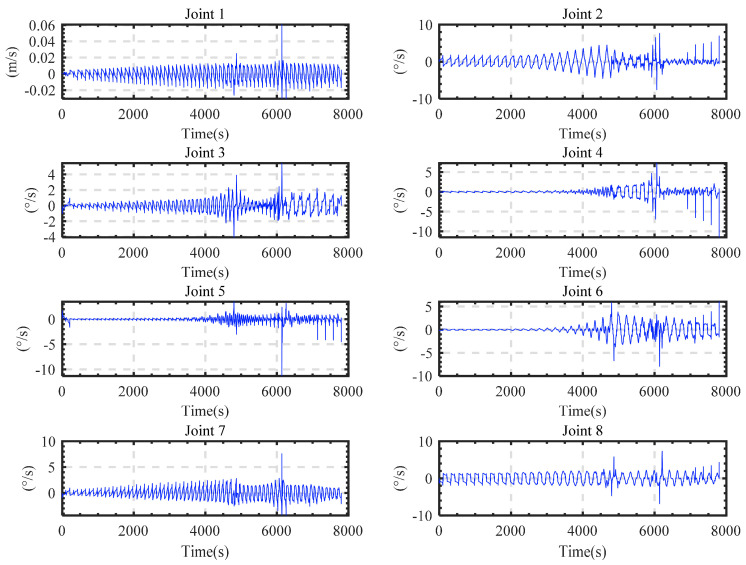
The velocity of each joint of the trajectory planned by the improved trajectory strategy.

**Figure 12:**
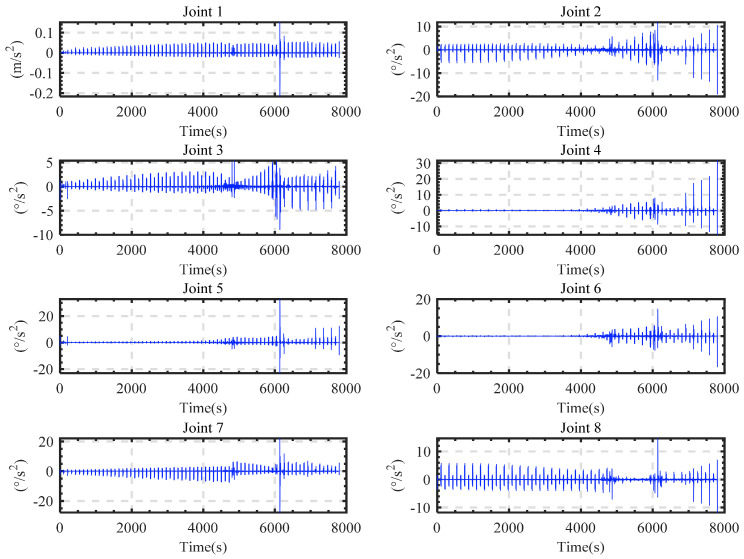
The acceleration of each joint of the trajectory planned by the improved trajectory strategy.

In order to verify the feasibility of the trajectory planning strategy proposed in this paper, the optimized manipulator in Section 2 was used as the manipulator model for the simulation experiments. The DH parameters of the optimized manipulator are calibrated to reduce the impact of assembly errors on the motion accuracy of the manipulator and to improve the smoothness of the trajectory. The improved trajectory planning strategy described in this paper was used to plan the scanning trajectory of the working sphere described in Section 3, and the motion trajectory planned with the improved strategy is shown in Figure 10, Figure 11 and Figure 12. Figure 10 shows the angle of each joint of the trajectory planned with the improved strategy. Figure 11 shows the velocity of each joint. Figure 12 shows the acceleration of each joint. It can be seen that the velocity and acceleration planned by the improved method are smooth.

**Figure 13:**
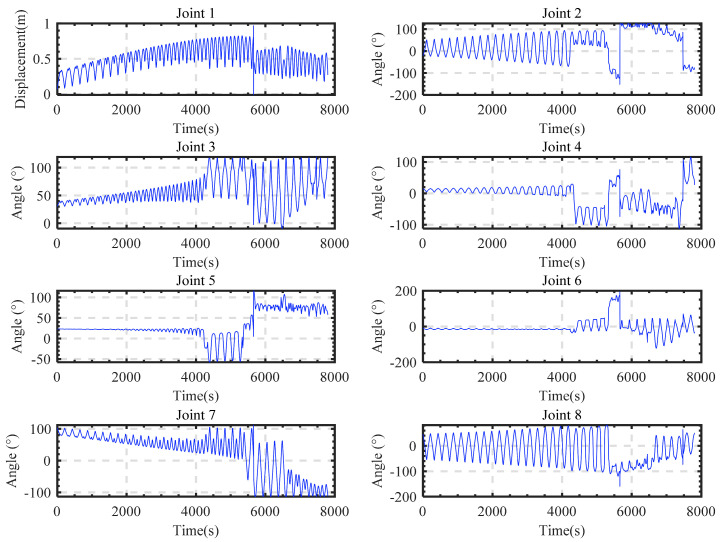
The angle of each joint of the trajectory planned by the general trajectory planning method.

**Figure 14:**
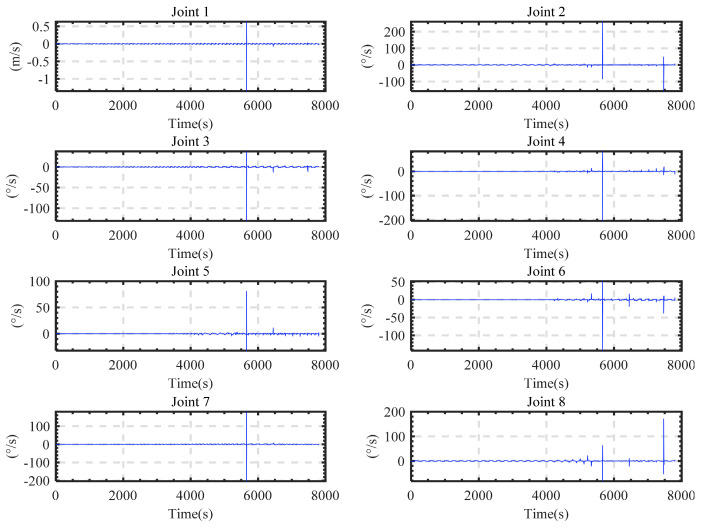
The velocity of each joint of the trajectory planned by the general trajectory planning method.

**Figure 15:**
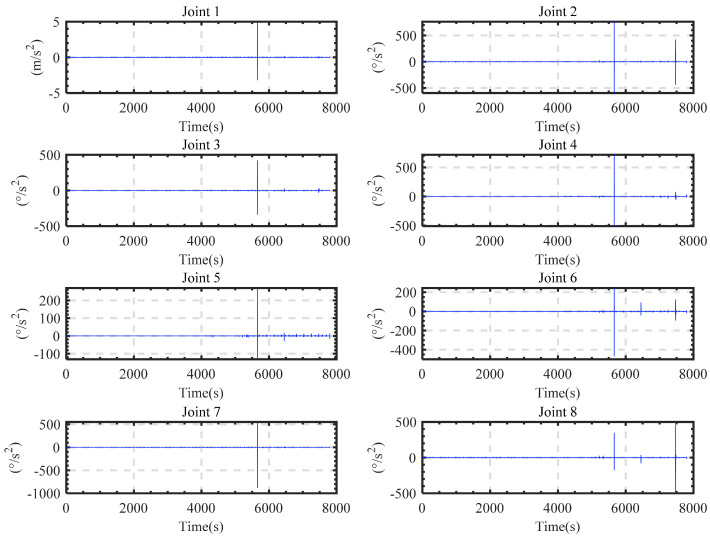
The acceleration of each joint of the trajectory planned by the general trajectory planning method.

At the same time, the inverse solution was solved using only the clamping weighted least-norm method and then interpolated using a quintic polynomial as a comparison. Figure 13, Figure 14 and Figure 15 show the results of the planning using the general method, where Figure 13 represents the angle of each joint of the trajectory, Figure 14 represents the joint velocity, and Figure 15 represents the joint acceleration. It can be seen from the graphs that there are several dramatic variations in the velocities and accelerations planned using the general method. A comparison of the two planned trajectories shows that the trajectories planned using the improved trajectory planning strategy are smoother. The feasibility of the improved trajectory planning strategy is demonstrated.

**Figure 16:**
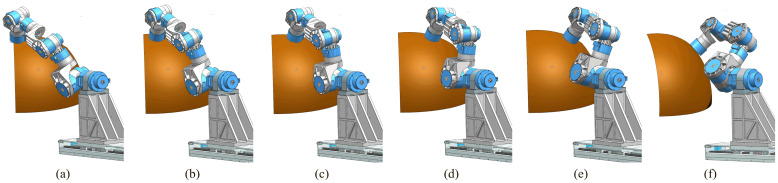
Simulation of manipulator motion along a section of trajectory. (**a**) is the starting point of the trajectory, (**b**–**e**) is the equally spaced midpoint of the trajectory, and (**f**) is the end point of the trajectory.

**Figure 17:**
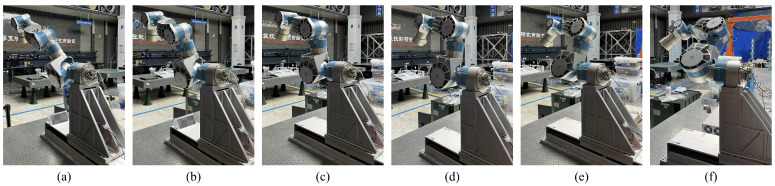
The actual process of manipulator motion along a section of trajectory. (**a**) is the starting point of the trajectory, (**b**–**e**) is the equally spaced midpoint of the trajectory, and (**f**) is the end point of the trajectory.

A section of the planned path is selected and run using motion simulation software and a real manipulator arm. The simulated motion is shown in Figure 16, and the motion of the manipulator is shown in Figure 17. From Figure 16 and Figure 17, it can be seen that the simulated motion is consistent with the actual motion of the manipulator, demonstrating the practicality of the trajectory planning method.

## 5. Discussion

In this paper, a redundant manipulator suitable for machining large curvature and large convex surfaces has been investigated, and the following conclusions are made:

(1) An eight-degree-of-freedom redundant manipulator is designed, and an improved multi-objective particle swarm optimization algorithm is used to optimize the configuration to obtain a suitable configuration.

(2) A trajectory planning strategy that combines the pincer-weighted minimum parametric method and the projected gradient method is proposed. The trajectory planning strategy is able to plan smooth machining trajectories for large-area curved surfaces.

(3) The simulation shows that the configuration of the eight-degree-of-freedom redundant manipulator can satisfy the requirements of large-area curved surface machining. Additionally, the trajectory planned by the improved strategy is smoother, and the experimental results prove the feasibility of the trajectory planning strategy proposed in this paper.

The optimization methods and trajectory strategies proposed in this paper for the large-surface machining manipulator still have some limitations, and the following aspects can be investigated in depth in future work. 

The configuration optimization process requires human judgment to determine a more accurate optimization range. Therefore, future research can be directed toward simplifying optimization methods and reducing human involvement. 

The trajectory planning takes longer because of the reverse planning step. Therefore, future research can be directed toward investigating more efficient singularity avoidance methods to improve the efficiency of trajectory planning. 

Although there is no mention of the machining of complex surfaces, the machining manipulator proposed in this paper is capable of machining complex surfaces. To machine complex surfaces, more detailed planning of the machining path is required. If we want to deal with different cases of workpieces, we can introduce visual inspection to determine the condition of the surface and then perform targeted trajectory planning, which is also an important future research direction [35].

Only the workspace and manipulator size were considered when optimizing the manipulator configuration. In the future, more performance parameters can be considered to make the manipulator perform better, while Boosting ensemble can be used as a reference to improve the speed and quality of optimization [36].

In addition, the proposed manipulator can be considered for the preparation and processing of novel materials, such as 3D-graphene and quantum dots [37,38]. The working space of the manipulator is large enough, and the planned trajectory is smooth, so the motion accuracy of the manipulator can be further improved to make it more versatile in the future.

## Figures and Tables

**Table 1:** D-H parameters.

Joint *i*	*α_i_*_−1_/(°)	*a_i_*_−1_/mm	*d_i_*/mm	*θ_i_*/(°)
1	−90	0	*d*_1_	−90
2	180	*L*_1_	0	*θ*_2_
3	90	0	0	*θ*_3_ + 90
4	90	0	*L*_2_	*θ*_4_
5	−90	0	0	*θ*_5_
6	90	0	*L*_3_	*θ*_6_
7	−90	0	0	*θ*_7_
8	90	0	*L*_4_	*θ*_8_ + 90

## Data Availability

The datasets generated and analysed during the current study are available from the corresponding author on reasonable request.
